# SNX27-Mediated Recycling of Neuroligin-2 Regulates Inhibitory Signaling

**DOI:** 10.1016/j.celrep.2019.10.096

**Published:** 2019-11-26

**Authors:** Els F. Halff, Blanka R. Szulc, Flavie Lesept, Josef T. Kittler

**Affiliations:** 1 Department of Neuroscience, Physiology, and Pharmacology, University College London, Gower Street, London WC1E 6BT, UK

**Keywords:** neuroligin, NLGN2, endocytosis, neuronal plasticity, E/I balance, GABA, retromer, SNX27, inhibitory signaling

## Abstract

GABA_A_ receptors mediate fast inhibitory transmission in the brain, and their number can be rapidly up- or downregulated to alter synaptic strength. Neuroligin-2 plays a critical role in the stabilization of synaptic GABA_A_ receptors and the development and maintenance of inhibitory synapses. To date, little is known about how the amount of neuroligin-2 at the synapse is regulated and whether neuroligin-2 trafficking affects inhibitory signaling. Here, we show that neuroligin-2, when internalized to endosomes, co-localizes with SNX27, a brain-enriched cargo-adaptor protein that facilitates membrane protein recycling. Direct interaction between the PDZ domain of SNX27 and PDZ-binding motif in neuroligin-2 enables membrane retrieval of neuroligin-2, thus enhancing synaptic neuroligin-2 clusters. Furthermore, SNX27 knockdown has the opposite effect. SNX27-mediated up- and downregulation of neuroligin-2 surface levels affects inhibitory synapse composition and signaling strength. Taken together, we show a role for SNX27-mediated recycling of neuroligin-2 in maintenance and signaling of the GABAergic synapse.

## Highlights

• Neuroligin-2 internalizes to endosomes and interacts with SNX27 and retromer
• Direct interaction between neuroligin-2 and SNX27 mediates neuroligin-2 recycling
• Modulation of synaptic neuroligin-2 levels affects inhibitory synapse composition
• SNX27-mediated recycling of neuroligin-2 regulates inhibitory signaling strength

## Graphical Abstract

**Figure:**
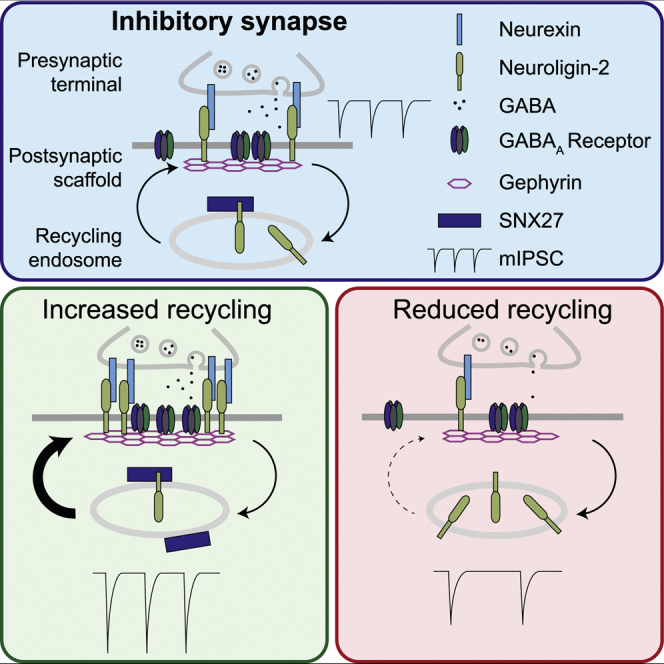

## Teaser

To maintain healthy brain function, neurons tightly regulate the balance between excitatory and inhibitory synaptic signaling. Neuroligin-2 is a postsynaptic adhesion protein important for inhibitory synapse function. In this article, Halff et al. describe how the protein SNX27 modulates synaptic levels of neuroligin-2, thereby regulating the strength of inhibitory signaling.

## Introduction

Synaptic inhibition is crucial for the correct operation of the brain by controlling the balance between excitation and inhibition of neurons (E/I balance). The major class of inhibitory synapses in the CNS contain gamma-aminobutyric acid (GABA) type A receptors (GABA_A_Rs). The postsynaptic adhesion molecule neuroligin-2 (NL2) plays a key role in the development and function of the GABAergic synapse and ensures its correct positioning opposite presynaptic terminals through interacting with presynaptic neurexins (Bemben et al., 2015, Nguyen et al., 2016, Südhof, 2017). NL2, by activating collybistin, drives clustering of a postsynaptic gephyrin scaffold, which stabilizes GABA_A_Rs at the synapse (Poulopoulos et al., 2009, Tyagarajan and Fritschy, 2014). To maintain the E/I balance in response to activity, synaptic signaling strength can be rapidly adapted through changing the number of receptors in the postsynaptic domain. This process may involve receptor endocytosis, followed either by recycling and reinsertion into the membrane, or lysosomal degradation for long-term downmodulation (Luscher et al., 2011, Arancibia-Cárcamo et al., 2009). A disruption in these mechanisms can lead to neurological disorders, including epilepsy and autism spectrum disorders (Smith and Kittler, 2010, Bozzi et al., 2018). Whereas the pathways involved in GABA_A_R endocytosis, degradation, and recycling have extensively been studied (Smith et al., 2012, Smith et al., 2014, Twelvetrees et al., 2010, Heisler et al., 2011, Eckel et al., 2015, Jacob et al., 2008), the molecular mechanisms that regulate NL2 trafficking and the amount of NL2 in synapses remain less well understood.

The brain-enriched protein sorting nexin 27 (SNX27) is an important endosome-cargo adaptor that, through association with retromer (a trimeric complex comprising VPS26, VPS29, and VPS35) and the retromer-associated Wiskott-Aldrich syndrome protein and SCAR homolog (WASH) complex, rescues cargo protein from lysosomal degradation and mediates their retrieval to the plasma membrane (Steinberg et al., 2013, Temkin et al., 2011, Gallon and Cullen, 2015). Among the SNX family of proteins, SNX27 uniquely contains a PDZ (postsynaptic density protein, *Drosophila* disc large tumor suppressor, and zonula occludens-1 protein) domain, through which it interacts with protein cargo containing a C-terminal PDZ-binding motif (Lunn et al., 2007). SNX27 cargo includes proteins involved in neuronal signaling, such as the β2 adrenergic receptor (Lauffer et al., 2010), AMPA receptors (Loo et al., 2014, Hussain et al., 2014, Temkin et al., 2017), and potassium channels (Lunn et al., 2007). Deficiencies in SNX27 function have been associated with Down syndrome (Wang et al., 2013) and epilepsy (Damseh et al., 2015).

A proteomics study identified NL2 as putative cargo for SNX27 (Steinberg et al., 2013); however, the functional implications of this interaction remain unclear. Here, we investigate trafficking of NL2 and show that SNX27 mediates plasma membrane retrieval of NL2. Through regulating NL2 surface availability, SNX27 function modulates inhibitory synapse composition and, ultimately, contributes to the regulation of inhibitory signaling.

## Results

### NL2 Internalizes to Recycling Endosomes and Interacts with SNX27 and Retromer

**Figure 1:**
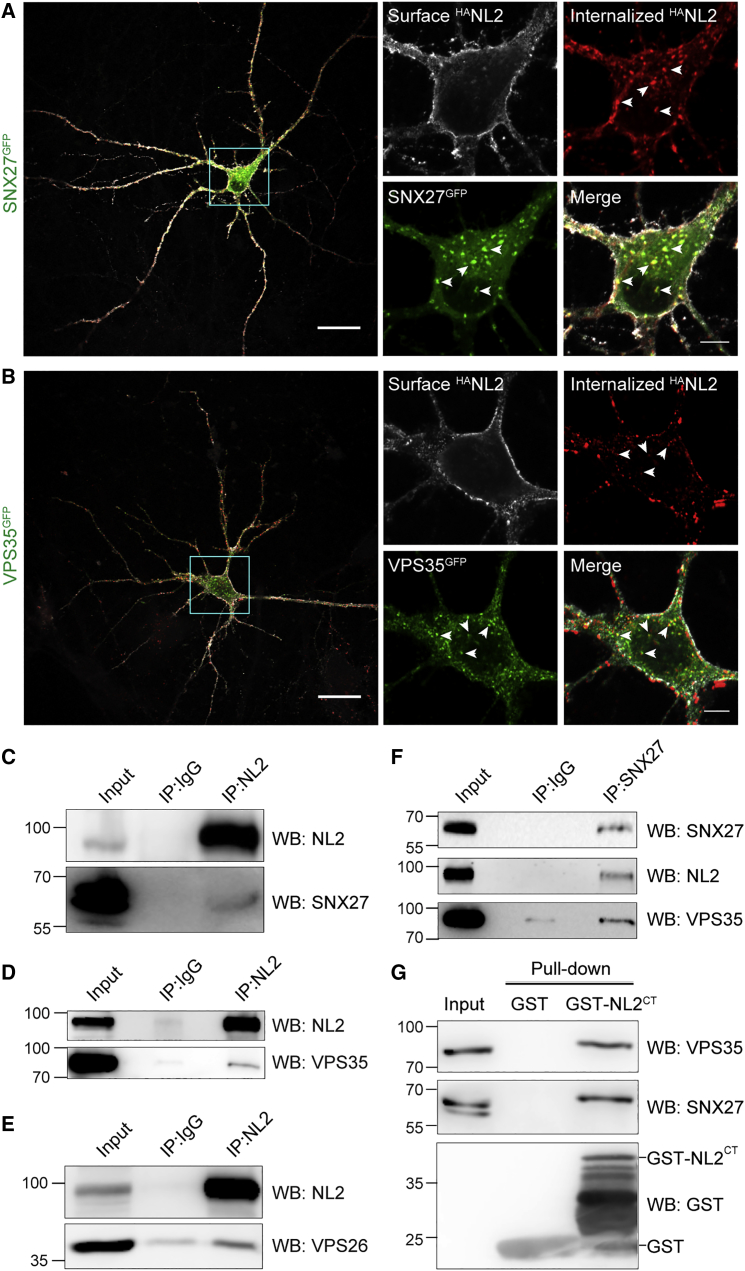
Internalized NL2 Co-localizes and Interacts with SNX27 and Retromer (A and B) Confocal images of antibody feeding in hippocampal neurons co-expressing ^HA^NL2 with either SNX27^GFP^ (A) or VPS35^GFP^ (B). Arrowheads show examples of co-localization. Scale bars, 25 μm (whole cell) and 5 μm (soma). (C–F) Western blots of co-immunoprecipitation from rat brain lysate via endogenous NL2 (C–E) showing interaction with endogenous SNX27 (C), VPS35 (D), and VPS26 (E) or via endogenous SNX27 (F) showing interaction with endogenous NL2 and VPS35. IP, immunoprecipitation. Numbers on the left indicate molecular weight in kDa. (G) Western blot of GST pull-down from rat brain lysate. See also Figure S1.

We hypothesized that surface levels of NL2 are regulated through endocytosis and recycling. To investigate trafficking of NL2, we used antibody feeding to follow the internalization of NL2 containing an HA tag at its extracellular N terminus (^HA^NL2). COS-7 cells were co-transfected with ^HA^NL2 and dsRed-tagged endocytic markers Rab5 or Rab11. ^HA^NL2 readily internalizes and co-localizes with both Rab5^dsRed^-positive early endosomes and Rab11^dsRed^-positive recycling endosomes (Figures S1A and S1B), indicating that it may be targeted for recycling. To verify a potential role for the recycling protein SNX27 in NL2 trafficking, we performed antibody feeding in HeLa cells and hippocampal neurons co-expressing ^HA^NL2 and GFP-tagged SNX27 (SNX27^GFP^). Internalized ^HA^NL2 co-localizes with SNX27^GFP^-positive puncta in both cell types (Figures 1A, S1C, and S1D). SNX27 associates with retromer to mediate cargo recycling (Gallon and Cullen, 2015). Accordingly, we find that internalized ^HA^NL2 also co-localizes with GFP-tagged retromer component VPS35 (VPS35^GFP^) in both HeLa cells and neurons (Figures 1B, S1E, and S1F), in agreement with a previous interaction study (Kang et al., 2014).

Next, we verified interaction between NL2 and SNX27/retromer at endogenous protein levels. Co-immunoprecipitation experiments from rat brain lysate demonstrate specific interaction of NL2 with SNX27 as well as retromer components VPS35 and VPS26 (Figures 1C–1F and S1G). To test whether this interaction involves the intracellular domain of NL2, we fused residue 699–835 to GST (GST-NL2^CT^; Figure S1H) and performed a GST-fusion protein pull-down in brain lysate. Both SNX27 and VPS35 interacted with GST-NL2^CT^ but not with GST alone (Figure 1G), confirming direct interaction between SNX27/retromer and the intracellular domain of NL2.

Taken together, these data show that NL2 can be endocytosed and, once internalized, co-localizes with SNX27 and retromer. Direct interaction between NL2 and SNX27/retromer suggests a role for this complex in trafficking of NL2.

### SNX27 Mediates Recycling of NL2 through PDZ-Ligand Interaction

**Figure 2:**
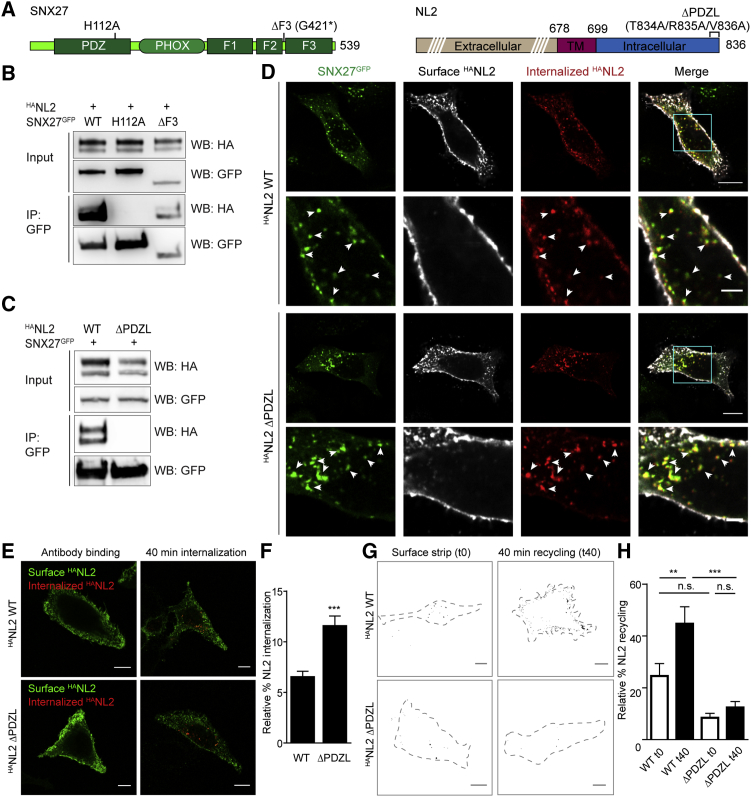
SNX27 Regulates Recycling of NL2 via PDZ-Ligand Interaction (A) Schematic representation of SNX27 (left) and NL2 (right), indicating the positions of mutations generated for this study. F1-3, FERM1-3; PDZL, PDZ-ligand; TM, transmembrane. (B and C) Western blots of co-immunoprecipitation from COS-7 cells co-expressing either WT ^HA^NL2 with WT or mutant SNX27^GFP^ (B) or WT SNX27^GFP^ with WT or mutant ^HA^NL2 (C). SNX27^GFP^ was pulled down using GFP-Trap beads. IP, immunoprecipitation. (D) Confocal images of antibody feeding in HeLa cells co-expressing ^HA^NL2 WT or ΔPDZL with SNX27^GFP^. Scale bars, 10 μm (whole cell) and 3 μm (zooms). Arrowheads show examples of co-localization. (E) Confocal images of antibody feeding in HeLa cells expressing ^HA^NL2 WT or ΔPDZL. Scale bar, 10 μm. (F) Quantification of relative ^HA^NL2 internalization in HeLa cells (n = 16; unpaired two-tailed t test). (G) Confocal images of antibody recycling in HeLa cells expressing ^HA^NL2 WT or ΔPDZL. To enable better visualization of extracellular ^HA^NL2 upon recycling, only the channel representing surface labeling is shown after thresholding and binarizing. Dashed lines indicate the outlines of the cell. Scale bar, 10 μm. (H) Quantification of relative ^HA^NL2 recycling in HeLa cells (n = 27; one-way ANOVA with Bonferroni’s correction). Values are mean ± SEM; n.s., non-significant. ^∗∗^p < 0.01 and ^∗∗∗^p < 0.001. See also Figure S2.

To test whether trafficking of NL2 involves its C-terminal PDZ-binding motif (or PDZ-ligand), we generated a mutant of ^HA^NL2 lacking this motif (^HA^NL2ΔPDZL), as well as a point mutation in the SNX27 PDZ domain that abolishes interaction with PDZ-binding motifs, SNX27^GFP^H112A (Hussain et al., 2014) (Figure 2A). We also created a non-related deletion mutant of SNX27 lacking the third FERM domain (SNX27^GFP^ΔF3); removal of this domain impedes recycling through loss of interaction with the WASH complex (Lee et al., 2016). These mutations in SNX27 did not affect its endosomal targeting, as shown by their vesicular staining and co-localization with endosomal marker EEA1 (early endosome antigen 1; Figure S2A). Co-immunoprecipitation in COS-7 cells expressing wild-type (WT) and mutant ^HA^NL2 and SNX27^GFP^ revealed that ^HA^NL2, while not pulled down by GFP alone (Figure S2B), interacted with both SNX27^GFP^WT and SNX27^GFP^ΔF3. The single point mutation in the SNX27 PDZ domain, however, completely abolished interaction with ^HA^NL2 (Figure 2B). Likewise, mutation of the PDZ-ligand in NL2 led to a complete loss of NL2-SNX27 interaction (Figure 2C). Thus, NL2 interacts with SNX27 via PDZ-ligand interaction.

Next, we investigated how NL2-SNX27 interaction directs trafficking of NL2. ^HA^NL2ΔPDZL, like ^HA^NL2WT, internalized to SNX27^GFP^-containing vesicles in both HeLa cells and neurons (Figures 2D and S2C–S2E), suggesting that endocytosis and initial sorting of NL2 are independent of SNX27. To characterize ^HA^NL2 internalization at endogenous levels of SNX27, we performed antibody feeding on HeLa cells overexpressing ^HA^NL2WT or ^HA^NL2ΔPDZL, but not SNX27^GFP^, and quantified the ratio between internal and total fluorescence as a relative measure for internalization (Figures 2E and 2F). We found a larger fraction of ^HA^NL2ΔPDZL to be localized intracellularly compared with ^HA^NL2WT, suggesting either increased internalization or decreased recycling of ^HA^NL2ΔPDZL. To quantify recycling, we allowed internalization of antibody-labeled ^HA^NL2, subsequently stripped the remaining surface-bound HA antibody, and then allowed for recycling of internalized antibody-labeled ^HA^NL2 (Figure 2G). This revealed that whereas ^HA^NL2WT re-appears at the membrane, ^HA^NL2ΔPDZL is impaired in its ability to recycle (Figure 2H). These results suggest that association between NL2 and SNX27 directs recycling of NL2.

### SNX27-Mediated Recycling of NL2 Enhances Inhibitory Signaling

**Figure 3:**
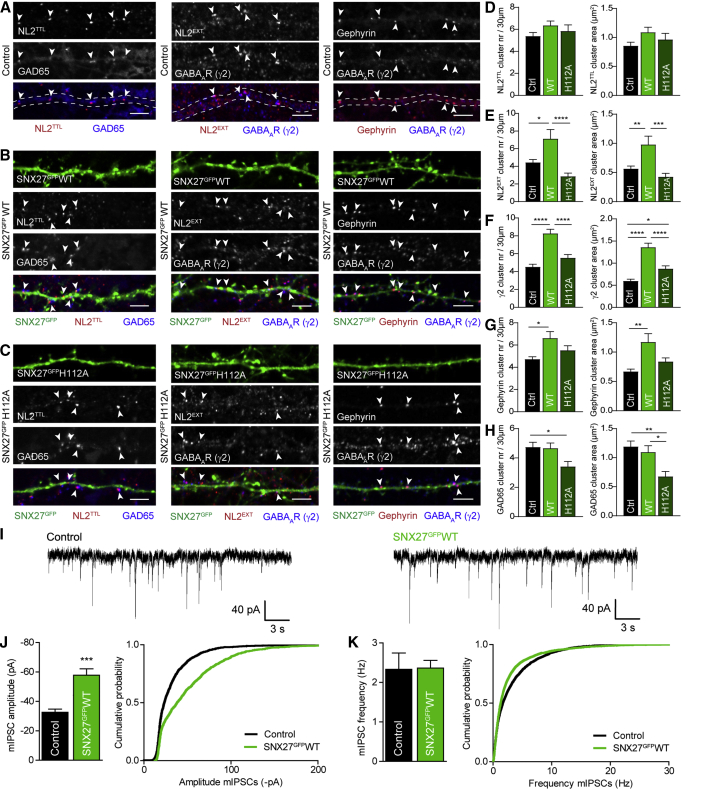
Overexpression of SNX27^GFP^WT but Not SNX27^GFP^H112A Increases Postsynaptic Clusters and Inhibitory Signaling (A–C) Confocal images of 30 μm dendritic sections of hippocampal neurons, mock transfected (control) (A) or overexpressing SNX27^GFP^WT (B) or SNX27^GFP^H112A (C). Neurons were stained for GAD65, total NL2 (NL2^TTL^), synaptic NL2 (NL2^EXT^), gephyrin, or the GABA_A_R γ2 subunit. Arrowheads show synaptic clusters. Dashed lines indicate the dendritic outline for mock-transfected cells (A). Scale bar, 4 μm. (D–H) Quantification of cluster number (left) and area (right) in hippocampal neurons either mock transfected (Ctrl) or overexpressing SNX27^GFP^WT (WT) or SNX27^GFP^H112A (H112A). Quantified are NL2^TTL^ (D) (n = 21, 19, and 19), NL2^EXT^ (E) (n = 24, 23, and 24), γ2 (F) (n = 35, 46, and 52), gephyrin (G) (n = 25, 34, and 26), and GAD65 (H) (n = 21, 20, and 17). (I) Representative traces of mIPSC patch-clamp recordings from hippocampal cultures, mock transfected (Control) or overexpressing SNX27^GFP^WT. (J and K) Pooled data (left) and cumulative probability plot (right) of mIPSCs amplitude (J) and frequency (K) (n = 13 and 22). Values are mean ± SEM. ^∗^p < 0.05, ^∗∗^p < 0.01, ^∗∗∗^p < 0.001, and ^∗∗∗∗^p < 0.0001, one-way ANOVA with Bonferroni’s correction (D–H) or unpaired two-tailed t test (J and K). See also Figure S3.

To investigate whether SNX27 also mediates recycling of endogenous NL2 in neurons, we transfected hippocampal cultures with either SNX27^GFP^WT or the mutant SNX27^GFP^H112A and assessed the effects on inhibitory synapse composition and signaling in comparison with mock-transfected cells. Both overexpressed SNX27^GFP^ constructs are widely distributed on endosomes throughout the cell (Figure S3A). We analyzed the number and size of inhibitory synapses by immunostaining for NL2, the presynaptic marker GAD65, and postsynaptic markers gephyrin or the GABA_A_R γ2 subunit (Figures 3A–3C). To distinguish between extracellular (synaptic) NL2 and total NL2, we separately stained with two different NL2 antibodies: one that detects an extracellular epitope and therefore, when incubated without permeabilization, only labels synaptic NL2 (NL2^EXT^) versus one that labels the intracellular domain of NL2 and thus, after permeabilization, detects the total pool of NL2 (NL2^TTL^). Overexpression of SNX27^GFP^WT, but not SNX27^GFP^H112A, led to a striking increase in the number and size of extracellular NL2 puncta, while total NL2 remained unaffected (Figures 3D and 3E). This suggests that overexpression of WT SNX27 results in NL2 redistribution, favoring synaptic localization of NL2, consistent with a role for SNX27 in NL2 recycling. The non-binding H112A mutant, however, did not facilitate recruitment of NL2 to the membrane but, in contrast, trended toward a decrease in synaptic NL2 clusters.

Simultaneously with increased surface NL2^EXT^, an increase in the number of synaptic GABA_A_Rs and gephyrin cluster number and size was observed with WT but not H112A SNX27^GFP^ overexpression (Figures 3F and 3G). Presynaptic terminals remained unaffected by SNX27^GFP^WT overexpression but appeared to be destabilized by SNX27^GFP^H112A (Figure 3H). Likewise, the number of presynaptic GAD65 clusters overlapping with postsynaptic γ2 clusters was not affected by SNX27^GFP^WT overexpression but reduced by overexpression of SNX27^GFP^H112A (Figure S3B). We postulate that this is related to the apparent reduction of synaptic NL2 in this condition. These data show that SNX27 enhances NL2 membrane retrieval, which in turn enables recruitment of gephyrin and GABA_A_Rs to the synapse.

To test the functional relevance of enhanced surface NL2 and GABA_A_Rs on inhibitory signaling, we performed whole-cell patch-clamp recordings of miniature inhibitory postsynaptic currents (mIPSCs) from hippocampal cultures overexpressing SNX27^GFP^WT in comparison with mock-transfected cells (Figures 3I–3K, S3C, and S3D). Overexpression of SNX27^GFP^WT resulted in an increase in mIPSC amplitude (Figure 3J) and corresponding total charge transfer (Figure S3C), which correlates with an increase in synaptic GABA_A_Rs (Figure 3F). The frequency of mIPSCs remained unaffected (Figure 3K). In a separate control experiment we recorded mIPSCs from hippocampal neurons overexpressing SNX27^GFP^H112A in comparison with mock-transfected cells (Figures S3E–S3I) and found no significant changes in mIPSC amplitude and frequency (Figures S3F and S3G), suggesting that the small increase in GABA_A_R cluster size upon SNX27^GFP^H112A overexpression (Figure 3F) has no functional consequence.

Taken together, these data show that SNX27-mediated retrieval of internalized NL2 to the synapse leads to enhanced recruitment of gephyrin and GABA_A_Rs, and increased inhibitory signaling.

### Reduced NL2 Recycling Interferes with Inhibitory Synaptic Function

**Figure 4:**
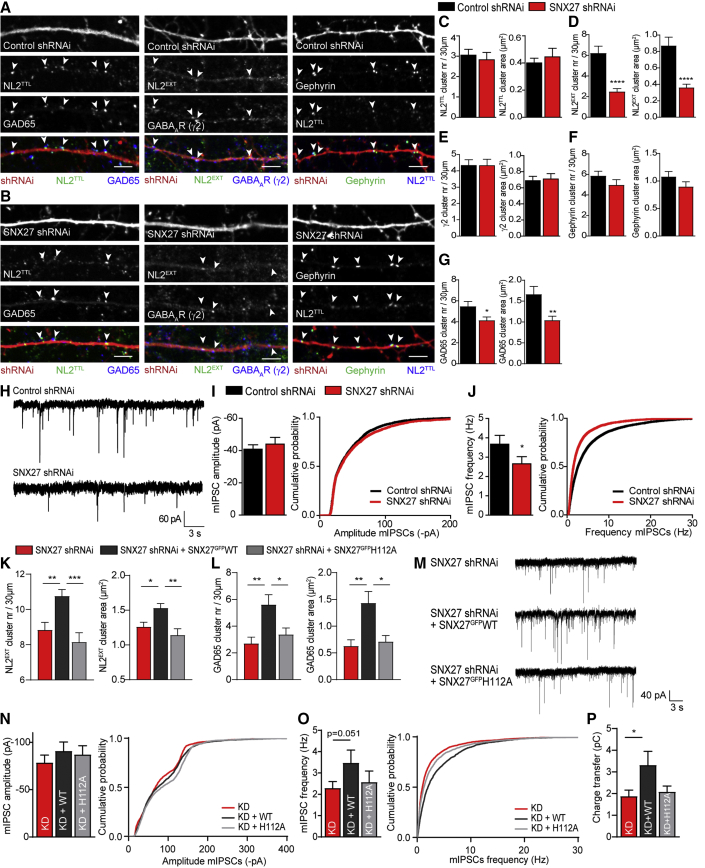
SNX27 Knockdown Decreases Synaptic NL2 and Disrupts Inhibitory Signaling (A and B) Confocal images of 30 μm dendritic sections of hippocampal neurons, overexpressing control shRNAi (A) or SNX27-specific shRNAi (B). Neurons were stained as in Figures 3A–3C. Arrowheads show synaptic clusters. Scale bar, 4 μm. (C–G) Quantification of cluster number (left) and area (right) in hippocampal neurons transfected as in (A) and (B). Quantified are NL2^TTL^ (C) (n = 19 and 17; cluster number, unpaired two-tailed t test; cluster area, Mann-Whitney test), NL2^EXT^ (D) (n = 36 and 37; Mann-Whitney tests), γ2 (E) (n = 20 and 19; unpaired two-tailed t tests), gephyrin (F) (n = 19 and 17; Mann-Whitney tests), and GAD65 (G) (n = 27; cluster number, Mann-Whitney test; cluster area, unpaired two-tailed t test). (H) Representative traces of mIPSC patch-clamp recordings from hippocampal cultures overexpressing control or SNX27-specific shRNAi. (I and J) Pooled data (left) and cumulative probability (right) of mIPSC amplitude (I) and frequency (J) (n = 23 and 20; unpaired one-tailed t tests). (K and L) Quantification of cluster number (left) and area (right) in hippocampal neurons transfected with SNX27-specific shRNAi alone (red) or combined with RNAi-resistant SNX27^GFP^WT (black) or SNX27^GFP^H112A (gray). Quantified are NL2^EXT^ (K) (n = 22, 23, and 19; one-way ANOVA with Bonferroni’s correction) and GAD65 (L) (n = 23, 22, and 28; Kruskal-Wallis test with Dunn’s correction). See Figures S4I and S4J for quantification of γ2 and gephyrin. (M) Representative traces of mIPSC patch-clamp recordings from hippocampal cultures overexpressing SNX27-specific shRNAi alone (KD) or combined with RNAi-resistant SNX27^GFP^WT (KD+WT) or SNX27^GFP^H112A (KD+H112A). (N and O) Pooled data (left) and cumulative probability (right) of mIPSC amplitude (N) and frequency (O) (n = 14, 14, and 13; unpaired one-tailed t tests). (P) Quantification of mIPSC charge transfer (unpaired one-tailed t tests). Values are mean ± SEM. ^∗^p < 0.05, ^∗∗^p < 0.01, ^∗∗∗^p < 0.001, ^∗∗∗∗^p < 0.0001. See also Figure S4.

Next, we transfected hippocampal cultures with SNX27-specific short hairpin RNAi (shRNAi) and confirmed efficient knockdown (KD) in comparison with control shRNAi (Figures S4A and S4B). We then analyzed the impact of interfering with NL2 recycling on the number and size of inhibitory synaptic clusters (Figures 4A–4G). Whereas total levels of NL2 were unaffected, we found a 60% decrease in synaptic NL2 clusters (Figures 4C and 4D), indicating that SNX27 KD results in redistribution of NL2 to intracellular pools. Interestingly, the number and size of GABA_A_R and gephyrin clusters remained unchanged (Figures 4E and 4F), but we observed a significant reduction in presynaptic GAD65 clusters (Figure 4G) and, consequently, in overlapping GAD65/γ2 clusters (Figure S4C). mIPSC recordings from hippocampal cultures expressing control or SNX27-specific shRNAi (Figures 4H–4J, S4D, and S4E) showed no change in mIPSC amplitude (Figure 4I), which concurs with a lack of change in surface GABA_A_Rs. In contrast, mIPSC frequency was significantly decreased (Figure 4J), consistent with a decrease in presynaptic terminals (Figure 4G).

To confirm that the reduction in synaptic NL2 clusters, GAD65 clusters, and mIPSC frequency is specific to the loss of SNX27, we performed KD and rescue experiments using RNAi-resistant SNX27^GFP^WT or SNX27^GFP^H112A and quantified synaptic clusters using immunostaining (Figures S4F–S4H) and inhibitory signaling by recording mIPSCs (Figures 4M–4P and S4L) as before. KD combined with SNX27^GFP^WT, but not SNX27^GFP^H112A, restored the number and size of synaptic NL2 clusters as well as GAD65 clusters (Figures 4K and 4L). We observed an increase in γ2 cluster area, reminiscent of the effect of SNX27 overexpression without KD, but γ2 cluster number, as well as gephyrin clusters and GAD65/γ2 overlap remained unaffected (Figures S4I–S4K). The amplitude of mIPSCs was unaffected by SNX27 KD, and likewise co-expression with rescue constructs did not alter the mIPSC amplitude (Figure 4N). However, we did observe a trend toward a rescue of mIPSC frequency upon WT but not H112A SNX27^GFP^ co-expression (p = 0.051; Figure 4O). Moreover, the total charge transfer, a parameter that reflects both the amplitude and frequency of miniature synaptic events, significantly increased upon rescue with WT but not H112A SNX27^GFP^ (Figure 4P), in agreement with the increased γ2 and GAD65 clusters in this condition.

Thus, we show that SNX27 KD leads to a loss of NL2 from the synapse, synaptic destabilization, and decreased inhibitory signaling. These effects can be rescued by reintroducing SNX27^GFP^WT. All data taken together, we provide evidence that SNX27 regulates surface availability of NL2 and, as a result, the stability and composition as well as signaling strength of the inhibitory synapse.

## Discussion

Neuroligins are well known for their role in regulating the development, function, and plasticity of synapses (Bemben et al., 2015, Varoqueaux et al., 2006). The mechanisms that direct the trafficking, and thereby alter synaptic levels, of neuroligins themselves, however, remain less well understood. In this study we investigated how surface levels of neuroligin-2 (NL2) are regulated and how this affects inhibitory signaling. We show that NL2 can be endocytosed and subsequently co-localizes and interacts with recycling protein SNX27 and retromer. SNX27 enables membrane retrieval of internalized NL2 through direct PDZ-ligand interaction. Finally, we show that SNX27-mediated modulation of NL2 surface availability contributes to the regulation of inhibitory synapse composition and signaling strength.

Our data support the following molecular model (Figure S4M). SNX27 activity enhances retrieval of NL2 to the synapse and consequently positively modulates postsynaptic gephyrin and GABA_A_R clustering, thereby enhancing inhibitory currents, consistent with the well-known role of NL2 in synapse maintenance (Varoqueaux et al., 2006). We speculate that this effect may be through the stabilization of GABA_A_Rs that were already present at extra-synaptic sites. Considering that GABA_A_R subunits do not possess a PDZ-binding motif and do not interact with SNX27 directly (Wang et al., 2013), and that SNX27 KD does not decrease synaptic GABA_A_Rs levels (Figure 4; Wang et al., 2013), we speculate that endocytosis and recycling of NL2 and GABA_A_Rs involve separate mechanisms. We furthermore propose that the reduced inhibitory signaling observed upon SNX27 KD and loss of NL2 at the synapse is due to reduced transsynaptic interactions, particularly between NL2 and neurexin (Dean et al., 2003, Shipman and Nicoll, 2012), consequently destabilizing the presynaptic terminal. Likewise, NL2 KD reduces presynaptic vGAT clusters (Chih et al., 2005).

The SNX27/retromer/WASH nexus has been characterized as machinery that rescues internalized cargo from lysosomal degradation (Lee et al., 2016). Indeed, while our manuscript was in revision, another study demonstrated a role for SNX27 in rescuing NL2 from lysosomal degradation (Binda et al., 2019), although that study did not assess its effect on inhibitory transmission. Using surface protein biotinylation, they demonstrate reduced NL2 surface levels upon SNX27 KD, consistent with our immunostaining data showing reduced synaptic NL2 upon SNX27 shRNAi (Figure 4D). Whereas they also report reduced total NL2 protein levels and gephyrin cluster numbers upon SNX27 KD, we found no change in total NL2 or gephyrin clusters in this condition (Figures 4C and 4F). A possible explanation for the difference is that we transfected and analyzed the neurons at a younger age (day *in vitro* [DIV] 7 and DIV 13, respectively). We speculate that at the neuronal age and in the time frame of our experiments, impaired NL2 recycling leads to a redistribution of NL2 from the surface to internal storage, whereas enhanced degradation only occurs at a later time point. In a similar way, the β1-adrenergic receptor was shown to be retained at the trans-Golgi network and protected from degradation upon internalization (Koliwer et al., 2015). In comparison with the slower process of degradation and *de novo* synthesis, membrane protein internalization and recycling enables more rapid modulation of protein surface levels, on a timescale of seconds, to respond to changes in the E/I balance (Luscher et al., 2011). Our data are thus consistent with a model in which internalized NL2 is stored in a “ready-to-go” intracellular pool, enabling rapid SNX27-mediated membrane reinsertion and upregulation of inhibitory signaling.

A major question yet to be answered is which physiological signals prompt enhanced endocytosis of NL2 and intracellular retainment, as well as the reverse mechanism leading to increased NL2 insertion by SNX27. It is tempting to speculate that these processes are determined by neuronal activity and thus that the modulation of synaptic NL2 levels plays a role in maintaining the E/I balance. In a recent study, Kang et al. (2014) identified a range of protein interactors of NL2, including regulators of endocytosis, trafficking, and phosphorylation. Although the functional roles of these proteins in modulating surface levels of NL2 remain to be characterized, this work and ours together are suggestive of a role for directed trafficking of NL2 in synaptic signaling.

Possible mechanisms that direct NL2 internalization versus recycling could include phosphorylation of the residues adjacent to its PDZ-ligand, as phosphorylation near these motifs has been shown to determine binding affinity between the SNX27 PDZ domain and its ligand (Clairfeuille et al., 2016). PDZ domain containing proteins play a dual role in synaptic function: on one hand, scaffold proteins such as PSD95 and other members of the MAGUK family stabilize transmembrane proteins at the postsynaptic domain via PDZ interaction (Meyer et al., 2004); NL2 itself is stabilized at the synapse by S-SCAM (Sumita et al., 2007). On the other hand, PDZ-ligand interactions are essential for trafficking in neuronal function (Bauch et al., 2014, Koliwer et al., 2015). Thus, regulation of affinity between a PDZ domain and its ligand may play an important role in modulating surface availability of synaptic proteins. Our data suggest that the PDZ-ligand is not required for *de novo* membrane insertion of NL2, as ^HA^NL2ΔPDZL still localizes to the cell membrane (Figure 2D) and interfering with SNX27-mediated NL2 recycling reduces but does not abolish inhibitory synapse formation (Figure 4). Accordingly, previous studies showed that the PDZ-ligand is dispensable for the synaptogenic properties of neuroligins (Shipman et al., 2011, Li et al., 2017).

Previously characterized SNX27 cargo includes G-protein-coupled receptors (Lauffer et al., 2010, Lin et al., 2015) and excitatory synaptic receptors (Loo et al., 2014, Hussain et al., 2014). Our study now provides evidence that SNX27 plays a role in regulating inhibitory signaling. Importantly, the PDZ-binding motif is conserved in other neuroligins, and SNX27 also interacts with and regulates protein levels of NL1 and NL3 (Binda et al., 2019). Specifically altered trafficking of NL3 may affect inhibitory synapse function (Davenport et al., 2019, Nguyen et al., 2016) and have an additional impact on inhibitory synapse modulation as assessed in our study. Future studies could include SNX27 KD in a neuroligin-null background combined with rescue of individual neuroligins to dissect specific effects of each neuroligin family member. Disrupted SNX27-dependent trafficking of both inhibitory and excitatory synaptic cargo may contribute to altered E/I balance in SNX27-associated neurological diseases, including Down syndrome and epilepsy (Wang et al., 2013, Damseh et al., 2015). Given that epilepsy has been widely ascribed to impaired synaptic inhibition (Gouzer et al., 2014, González, 2013), it would be interesting to investigate whether disrupted NL2 recycling coincides with an increased chance of epileptiform activity.

All in all, we have shown that SNX27-mediated modulation of synaptic NL2 levels is an important mechanism in the regulation of inhibitory signaling.

## STAR★Methods

### Key Resources Table

**Table:** 

REAGENT or RESOURCE	SOURCE	IDENTIFIER
**Antibodies**		

Rabbit IgG control	ThermoFisher Scientific	Cat# 10500C; RRID: AB_2532981
Mouse IgG control	ThermoFisher Scientific	Cat# 10400C; RRID: AB_2532980
Mouse-anti-EEA1	BD Transduction Labs	Cat# 610457; Clone# 14/EEA1; RRID: AB_397830
Guinea Pig-anti-GABA_A_R-ɣ2	Synaptic Systems	Cat# 224 004; RRID: AB_10594245
Mouse-anti-GAD65 (supernatant)	Neuromab	Cat# 73-508; Clone#L127/12; RRID: AB_2756510
Mouse-anti-Gephyrin	Synaptic Systems	Cat# 147 011; Clone# mAb7a; RRID: AB_887717
Rabbit-anti-GFP	SantaCruz	Cat# sc-8334; RRID: AB_641123
Rat-anti-GFP	Nacalai Tesque	Cat# 04404-84; RRID: AB_10013361
Mouse-anti-GST (supernatant)	Neuromab	Cat# 75-148; clone# N100/13; RRID: AB_10671817
Mouse-anti-HA-tag	Produced and purified in house from hybridoma cells. Vendor: James Trimmer, UC Davis	Clone# 12CA5; RRID: AB_2532070
Rabbit-anti-Neuroligin2	Alomone Labs	Cat# ANR-036; RRID: AB_2341007
Rabbit-anti-Neuroligin2	Synaptic Systems	Cat# 129-202; RRID: AB_993011
Mouse-anti-SNX27	Abcam	Cat# ab77799; RRID: AB_10673818
Rabbit-anti-SNX27	Atlas antibodies	Cat# HPA045816 (discontinued)
Rabbit-anti-VPS26	Abcam	Cat# ab181352; RRID: AB_2665924
Rabbit-anti-VPS35	Abcam	Cat# ab97545; RRID: AB_10696107
Rabbit-anti-VPS35	Atlas antibodies	Cat# HPA040802; RRID: AB_2677142
Goat-anti-rabbit IgG (H+L), HRP	Jackson ImmunoResearch	Cat# 111-035-003; RRID: AB_2313567
Goat-anti-mouse IgG (H+L), HRP	Jackson ImmunoResearch	Cat# 115-035-003; RRID: AB_10015289
Goat anti-mouse IgG (light chain specific), HRP	Jackson ImmunoResearch	Cat# 115-035-174; RRID: AB_2338512
Goat-anti-mouse AlexaFluor 405	ThermoFisher Scientific	Cat# A-31553; RRID: AB_221604
Donkey-anti-mouse AlexaFluor 488	Jackson ImmunoResearch	Cat# 715-545-151; RRID: AB_2341099
Donkey-anti-rabbit AlexaFluor 488	ThermoFisher Scientific	Cat# A-21206; RRID: AB_2535792
Donkey-anti-rat AlexaFluor 488	ThermoFisher Scientific	Cat# A-21208; RRID: AB_141709
Goat-anti-mouse AlexaFluor 555	ThermoFisher Scientific	Cat# A-21424; RRID: AB_141780
Goat-anti-rabbit AlexaFluor 555	ThermoFisher Scientific	Cat# A-21430; RRID: AB_2535851
Goat-anti-guinea pig AlexaFluor 647	ThermoFisher Scientific	Cat# A-21450; RRID: AB_2535867
Donkey-anti-mouse AlexaFluor 647	ThermoFisher Scientific	Cat# A-31571; RRID: AB_162542
Donkey-anti-rabbit AlexaFluor 647	ThermoFisher Scientific	Cat# A-31573; RRID: AB_2536183

**Bacterial and Virus Strains**		

One Shot TOP10 Chemically Competent *E.coli*	Invitrogen	Cat# C404010
BL21(DE3) One Shot Chemically Competent *E.Coli*	Invitrogen	Cat# C600003

**Chemicals, Peptides, and Recombinant Proteins**		

Hank’s Buffered Salt Solution (HBSS)	GIBCO	Cat# 14180046
1M HEPES buffer	GIBCO	Cat# 15630080
Minimal Essential Medium (MEM)	GIBCO	Cat# 31095029
Heat inactivated Horse Serum (HRS)	GIBCO	Cat# 26050088
Sodium pyruvate	GIBCO	Cat# 11360070
Glucose	GIBCO	Cat# A2494001
Neurobasal medium	GIBCO	Cat# 21103049
B-27	GIBCO	Cat# 17504044
GlutaMAX	GIBCO	Cat# 35050061
DMEM (high glucose)	GIBCO	Cat# 41965039
Fetal Bovine Serum	GIBCO	Cat# 10082147
Penicillin/Streptomycin	GIBCO	Cat# 15140122
2.5% Trypsin	GIBCO	Cat# 15090046
DNase	Sigma-Aldrich	Cat# DN-25
Poly-L-lysine (PLL)	Sigma-Aldrich	Cat# P6282-5MG
Lipofectamine-2000	Invitrogen	Cat# 11668027
CsCl	Sigma-Aldrich	Cat# C4036
QX314 Br	Sigma-Aldrich	Cat# L5783
NBQX	Abcam	Cat# ab120046
APV	Abcam	Cat# ab120003
Tetrodotoxin citrate (TTX)	Tocris	Cat# 1078
IPTG	Melford	Cat# 367-93-1
PMSF	AppliChem	Cat# A0999,0025
Antipain	Peptide	Cat# 4062
Pepstatin	Peptide	Cat# 4397
Leupeptin	Peptide	Cat# 4041
Glutathione Sepharose 4B	GE Healthcare	Cat# 17075601
Protein A Sepharose	Generon	Cat# PC-A25
GFP-Trap	Chromotek	Cat# gta-100
Luminate Crescendo Western HRP substrate	Milipore	Cat# WBLUR0500
ProLong Gold antifade reagent	Invitrogen	Cat# P36930

**Critical Commercial Assays**		

BioRad protein assay	BioRad	Cat# PI-23225
Gateway™ LR Clonase™ Enzyme Mix	ThermoFisher Scientific	Cat# 11791019
In-Fusion® HD Cloning Plus	Takara	Cat# 638909

**Experimental Models: Cell Lines**		

COS-7	ATCC	Cat# CRL-1651; RRID: CVCL_0224
HeLa	ATCC	Cat# CRM-CCL-2; RRID: CVCL_0030

**Experimental Models: Organisms/Strains**		

Wild-type Sprague-Dawley rats	Charles River	N/A

**Oligonucleotides**		

Subcloning mSNX27 forward primer: gatctcgagctcaagctt atggcggacgaggacgg	This paper	N/A
Subcloning mSNX27 reverse primer: catggtggcgaccggtgg ggtggccacatccctctg	This paper	N/A
Subcloning mSNX27-ΔF3 reverse primer: catggtggcgacc ggtgggccctcgcaggtccttag	This paper	N/A
Mutagenesis to create RNAi-resistant mSNX27, forward primer: gggcagctggagaaccaagtgatcgcattcgaatgggatga gatgc	Hussain et al., 2014	N/A
Mutagenesis to create RNAi-resistant mSNX27, reverse primer: gcatctcatcccattcgaatgcgatcacttggttctccagctgccc	Hussain et al., 2014	N/A
Mutagenesis to create mSNX27-H112A, forward primer: gagggggcgacagccaagcaggtggtgg	This paper	N/A
Mutagenesis to create mSNX27-H112A, reverse primer: ccaccacctgcttggctgtcgccccctc	This paper	N/A
Subcloning GST-NL2^CT^, forward primer: catcatggatccta caagcgggaccggcgcc	This paper	N/A
Subcloning GST-NL2^CT^, reverse primer: catcatctcgagcta tacccgagtggtggagtg	This paper	N/A

**Recombinant DNA**		

pNICE-NL2(-)	Peter Scheiffele (Chih et al., 2006)	Cat# Addgene 15246
pNICE-NL2(-)-ΔPDZL	This paper	N/A
pCMV6-Entry-mSNX27	OriGene	Cat# MR218832
pEGFP-N1-mSNX27	This paper	N/A
pEGFP-N1-mSNX27-ΔF3	This paper	N/A
pEGFP-N1-mSNX27-H112A	This paper	N/A
pEGFP-N1-mSNX27 RNAi-resistant	This paper	N/A
pEGFP-N1-mSNX27-H112A RNAi-resistant	This paper	N/A
pDONR223-VPS35	Lynda Chin (Scott et al., 2009)	Cat# Addgene 21689
pDEST-eGFP-C1-VPS35	This paper	N/A
pDsRed-Rab5	Richard Pagano (Sharma et al., 2003)	Cat# Addgene 13050
pDsRed-Rab11	Richard Pagano (Choudhury et al., 2002)	Cat# Addgene 12679
pEGFP-N1	Clontech	Cat# 6085-1
pDEST-eGFP-C1	Robin Shaw (Hong et al., 2010)	Cat# Addgene 31796
pGEX4T3	GE Healthcare	Cat# 28954552
pGEX4T3-NL2^CT^	This paper	N/A
pSuper-dsRed control	Kumiko Ui-Tei (Nishi et al., 2013)	Cat# Addgene 42053
pSuper-dsRed SNX27 shRNAi	This paper, following method as published (Hussain et al., 2014)	N/A

**Software and Algorithms**		

Fiji/ImageJ	National Institutes of Health	https://imagej.net/Welcome RRID: SCR_003070
Metamorph	Molecular Devices	N/A
ZEN LSM	Zeiss	N/A
GraphPad Prism	GraphPad Software	N/A
Clampfit	Molecular Devices	N/A

### Lead Contact and Materials Availability

Further information and requests for resources and reagents should be directed to and will be fulfilled by the Lead Contact, Josef T. Kittler (j.kittler@ucl.ac.uk). Plasmids generated in this study are available from the Lead Contact upon request.

### Experimental Model and Subject Details

#### Animals

All procedures for the care and treatment of animals were in accordance with the Animals (Scientific Procedures) Act 1986, and had full Home Office ethical approval. Animals were maintained under controlled conditions (temperature 20 ± 2°C; 12h light-dark cycle), were housed in conventional cages and had not been subject to previous procedures. Food and water were provided *ad libitum*. Wild-type E18 Sprague-Dawley rats were generated as a result of wild-type breeding; embryos of either sex were used for generating primary neuronal cultures.

#### Primary Hippocampal Culture and transfection

Cultures of hippocampal neurons were prepared from E18 WT Sprague-Dawley rat embryos of either sex as previously described (Muir and Kittler, 2014, Davenport et al., 2019). In brief, rat hippocampi were dissected from embryonic brains in ice-cold HBSS (GIBCO) supplemented with 10mM HEPES. Dissected hippocampi were incubated in the presence of 0.25% trypsin and 5 Units/ml DNase for 15 mins at 37°C, washed twice in HBSS with HEPES, and triturated to a single cell suspension in attachment media (MEM (GIBCO) containing 10% horse serum, 10 mM sodium pyruvate, and 0.6% glucose) using a fire-polished glass pasteur pipette. Dissociated cells were plated on poly-L-lysine coated coverslips in attachment media at a density of 5x10^5^ cells/6 cm dish. After 6h serum-containing medium was replaced with Neurobasal medium (GIBCO) containing 2% B-27 (GIBCO), 2 mM glutaMAX (GIBCO), 100 U/ml penicillin and 100 μg/ml streptomycin. Cultures were maintained at 37°C in humidified atmosphere with 5% CO_2_. Neurons were transfected using Lipofectamine 2000 (Invitrogen) at DIV10 for antibody feeding, or at DIV7 for cluster analysis and electrophysiology, and maintained until DIV13-14.

#### COS-7 and HeLa Cell culture and transfection

COS-7 and HeLa cells (ATCC) were maintained in DMEM (GIBCO), supplemented with 10% heat-inactivated fetal bovine serum, 100 U/ml penicillin and 100 μg/ml streptomycin, at 37°C in humidified atmosphere with 5% CO_2_. Cells were transfected 24h before further processing using the Amaxa Nucleofector® device (Lonza) following the manufacturer’s protocol.

#### Bacterial cell culture

OneShot TOP10 *E.coli* (used for standard cloning) and BL21(DE3) OneShot *E.Coli* (used for recombinant production of GST protein) were cultured in Luria broth medium according to standard protocols.

### Method Details

#### DNA Constructs

Murine SNX27 in pCMV6-Entry vector was purchased from Origene (United States) and subcloned into pEGFP-N1 (Clontech) via the *Hind*III and *Age*I sites using In-Fusion cloning (Clontech) and removal of the Stop codon (forward primer gatctcgagctcaagcttatggcggacgaggacgg; reverse primer catggtggcgaccggtggggtggccacatccctctg). The ΔF3 mutant was created via the same method, using a reverse primer that eliminates the C-terminal region (sequence: catggtggcgaccggtgggccctcgcaggtccttag). The H112A mutant and RNAi-resistant constructs of SNX27 WT and H112A were created using the Quickchange method (Agilent) (WT forward primer: gggcagctggagaaccaagtgatcgcattcgaatgggatgagatgc; reverse primer: gcatctcatcccattcgaatgcgatcacttggttctccagctgccc. H112A forward primer: gagggggcgacagccaagcaggtggtgg; reverse primer: ccaccacctgcttggctgtcgccccctc.).

HA-tagged murine NL2 lacking the splice A insert (pNICE-NL2(-)) was a gift from Peter Scheiffele (Addgene plasmid #15246; Chih et al., 2006). ^HA^NL2-ΔPDZL was generated using this NL2 cDNA as a template, where subsequent mutagenesis was performed by DNAExpress (Canada). To create GST-NL2^CT^, a PCR product of the intracellular domain flanked by BamHI and *Xho*I restriction sites was created (forward primer catcatggatcctacaagcgggaccggcgcc; reverse primer catcatctcgagctatacccgagtggtggagtg) and subcloned into pGEX4T3 (GE Healthcare).

pDONR223-VPS35 was a gift from Lynda Chin (Addgene plasmid #21689; Scott et al., 2009). VPS35 was subcloned into pDEST-eGFP-C1 (Addgene plasmid #31796; Hong et al., 2010) using Gateway LR Clonase II (ThermoFisher). Rab5-dsRed and Rab11-dsRed were obtained from Addgene (plasmid #13050 and #12679, respectively; Sharma et al., 2003, Choudhury et al., 2002). pSuper-dsRed was a gift from Kumiko Ui-Tei (Addgene plasmid #42053; Nishi et al., 2013). A pSuper vector containing SNX27 shRNAi was created as previously described (Hussain et al., 2014). The shRNAi sequence targets a sequence that is identical in rat and murine SNX27. This vector simultaneously expresses dsRed to enable identification of transfected cells.

#### Preparation of GSTfusion protein

GST fusion proteins were produced as described (Kittler et al., 2000). In brief, BL21 *E.Coli* containing empty pGEX4T3 or pGEX4T3-NL2^CT^ were grown in Luria Broth until an OD600 of 0.5-0.6. Protein production was induced by addition of 1mM IPTG, after which cells were grown for an additional 3h. Cells were harvested by centrifugation for 30 min at 4°C, washed in buffer containing 50 mM Tris pH 8.0, 25% sucrose, 10 mM EDTA, and pelleted by centrifugation for 30 mins at 4°C. Cells were then lysed by sonication in buffer containing 1% Triton X-100, 10 mM Tris pH 7.4, 1 mM EDTA, 1 mM DTT, 1 mM PMSF, and antipain, pepstatin and leupeptin at 10 μg/ml. Lysates were further incubated for 30 mins upon addition of 12.5 mM HEPES pH 7.6, 75 mM KCl, 125 mM EDTA, 12.5% glycerol, and then spun for 1h at 12,000x*g* and 4°C. GST protein was purified by adding Glutathione Sepharose 4B beads (GE Healthcare) and incubating for 2h at 4°C. Beads were washed and stored at 4°C in buffer containing 20 mM HEPES pH 7.6, 100 mM KCl, 0.2M EDTA, 20% glycerol, 1 mM DTT, and 1 mM PMSF.

#### GST pull-down and co-immunoprecipitation

Rat brain lysate was obtained from adult female WT Sprague-Dawley rats. A whole brain, excluding the cerebellum, was homogenized on ice in lysis buffer (50 mM HEPES pH 7.5, 0.5% Triton X-100, 150 mM NaCl, 1 mM EDTA, 1 mM PMSF with antipain, pepstatin and leupeptin at 10 μg/ml) and then left to rotate for 1h at 4°C. Membranes were pelleted by ultracentrifugation at 38,000x*g* for 40 mins at 4°C. Protein content of the supernatant was assayed by BioRad protein assay. For co-immunoprecipitations from brain lysate, 4 mg of brain lysate was incubated overnight at 4°C with rotation with 1 μg Rabbit IgG (ThermoFisher, 10500C) or Rabbit-anti-NL2 (Alomone Labs, ANR-036), or with 5 μg Mouse IgG (ThermoFisher, 10400C) or Mouse-anti-SNX27 (Abcam, ab77799). Complexes were precipitated with 15 μL of 50% protein A Sepharose bead slurry (Generon) for 2h at 4°C. For GSTpull-downs, 4 mg of brain lysate was incubated with 30 μg of GST or GST-NL2^CT^ fusion protein attached to Glutathione Sepharose 4B beads (GE Healthcare) for 2h at 4°C with rotation. For co-immunoprecipitations from COS-7 cells, transfected cells were lysed on a dish in lysis buffer by scraping. Cell lysates were left to rotate at 4°C for 1h. Membranes were pelleted by centrifugation at 14,000x*g* for 10 mins at 4°C. Supernatants were then incubated with 6 μL of a 50% GFP-Trap bead slurry (Chromotek) for 2h at 4°C. All beads were washed 3 times in lysis buffer before resuspending in sample buffer, and then analyzed by SDS-PAGE and western blotting.

#### Western blotting

Protein samples were separated by standard Laemli SDS-PAGE on 9% Tris-Glycine gels and transferred onto nitrocellulose membrane (GE Healthcare Bio-Sciences). Membranes were blocked for 1h in milk (PBS, 0.1% Tween, 4% milk), and then incubated overnight at 4°C with shaking in primary antibodies diluted in milk (1:1000 Rabbit-anti-Neuroligin2 (Synaptic Systems 129-202), 1:100 Rabbit-anti-VPS35 (Atlas antibodies, HPA040802, Figure 1G), 1:500 Rabbit-anti-VPS35 (Abcam, ab97545, Figures 1D and 1F), 1:2000 Rabbit-anti-VPS26 (Abcam, ab181352), 1:100 Rabbit-anti-SNX27 (Atlas antibodies, HPA045816, Figures 1C and 1G), 1:100 Mouse-anti-SNX27 (Abcam, ab77799, Figure 1F), 1:200 Mouse-anti-HA-tag (supernatant, clone 12CA5, produced in house), 1:500 Mouse-anti-GST (Neuromab, N100/13) or 1:500 Rabbit-anti-GFP (SantaCruz, sc-8334)). Blots were then incubated with the appropriate HRP-conjugated secondary antibody for 1h at room temperature and developed using Luminate Crescendo Western HRP substrate (Milipore). Signal was detected using an ImageQuant LAS4000 mini (GE Life Sciences).

#### Immunocytochemistry

For regular immunostaining, COS-7 and HeLa cells were fixed 24h post transfection, and cultured neurons were fixed at DIV13-14, for 7 mins in 4% PFA (PBS, 4% paraformaldehdye, 4% sucrose, pH 7). For surface labeling, cells were incubated in block solution (PBS, 10% horse serum, 0.5% BSA) for 10 mins, and then stained for 1h at RT with primary antibody diluted in block solution (1:100 Rabbit-anti-NL2 (Alomone Labs, ANR-036), 1:500 Guinea Pig-anti-γ2 (Synaptic Systems, 224-004)). Cells were then permeabilised in block solution supplemented with 0.2% Triton X-100, and incubated for 1h at RT with the appropriate primary antibodies (1:500 Rabbit-anti-Neuroligin2 (Synaptic Systems, 129-203), 1:500 mouse-anti-gephyrin (Synaptic Systems 147-011, clone mAb7a), 1:100 Mouse-anti-GAD65 (supernatant, Neuromab, L127/12), 1:1000 Rat-anti-GFP (Nacalai Tesque, 04404-84), 1:500 Mouse-anti-EEA1 (BD Transduction Labs, clone 14/EEA1)), followed by 45-60 mins incubation with the appropriate Alexa-fluorophore conjugated secondary antibody (goat-anti-mouse Alexa405 or donkey-anti-mouse Alexa488 or goat-anti-mouse Alexa555; donkey-anti-rabbit Alexa488 or goat-anti-rabbit Alexa555 or donkey-anti-rabbit Alexa-647; goat-anti-guinea pig Alexa647; donkey-anti-rat Alexa488). Finally, coverslips were mounted onto glass slides using ProLong Gold antifade reagent (Invitrogen).

Antibody feeding was performed as described (Arancibia-Cárcamo et al., 2009), and where necessary the protocol was adapted. Transfected COS-7 cells and HeLa cells were incubated for 15 mins at 12°C with 1:50 Mouse-anti-HA in serum-free DMEM supplemented with 10 mM HEPES pH 7.6. Unbound antibody was removed by washing in serum-free DMEM, before internalisation for 40 mins at 37°C. Where appropriate to visualize recycling, surface-bound antibody was stripped using acid wash (150 mM NaCl, 50 mM HAc), and internalised protein was allowed to recycle back to the membrane for 40 mins at 37°C. Antibody feeding in neurons (DIV13-14) was performed by adding 1:50 Mouse-anti-HA directly to neuronal maintenance medium and allowing internalisation for 40 mins at 37°C. Neurons were washed twice in medium before fixation and blocking. To distinguish between surface and internal ^HA^NL2, fixed cells were first incubated with 1:300 donkey-anti-mouse Alexa647 in block solution (PBS, 10% horse serum, 0.5% BSA) without detergent. Cells were then permeabilised in block solution supplemented with 0.2% Triton X-100 for 10 mins and incubated for 1h at RT with 1:1000 Rat-anti-GFP (Nacalai Tesque, 04404-84) to visualize SNX27 where applicable. Cells were then stained for 45 mins with 1:500 donkey-anti-rat Alexa488 and goat-anti-Mouse-Alexa-555, and finally mounted using ProLong Gold antifade reagent (Invitrogen).

#### Image acquisition and analysis

Confocal images were acquired on a Zeiss LSM700 upright confocal microscope using a 63x oil objective (NA: 1.4) and digitally captured using ZEN LSM software (version 2.3; Zeiss), with excitation at 405 nm for Alexa-Fluor405, 488 nm for GFP and Alexa-Fluor488, 555 nm for Alexa-Fluor555, and 647 nm for Alexa-Fluor647 conjugated secondary antibodies. Pinholes were set to 1 Airy unit creating an optical slice of 0.57 μm. For cultured neurons, a whole-cell stack was captured using a 0.5x zoom. Per neuron 3 sections of dendrite, ∼50 μm from the soma, were imaged with a 3.4x zoom (equating to 30 μm length of dendrite). For antibody feeding, settings were adjusted to ensure channels were not saturated. Acquisition settings and laser power were kept constant within experiments.

Cluster analysis was performed as described (Smith et al., 2014, Davenport et al., 2017). In brief, a suitable threshold was selected for each channel using Metamorph software (version 7.8; Molecular Devices) and applied to all images within the same dataset. Clusters with an intensity above this threshold and size of at least 0.05 μm^2^ were quantified. Quantification was performed on 5-8 cells per experiment.

For quantification of antibody feeding, images of surface and internal staining were thresholded to generate a mask, which was then used to measure total intensity of surface and internal signal in the original image. The ratio between internal and total (surface plus internal) intensity was calculated as a relative measure for internalisation; a relative measure for recycling was obtained by calculating the ratio between surface and total (surface plus internal) intensity.

Pearson’s correlation coefficient for co-localization, R, was determined using the co-localization tool Coloc2 in ImageJ. For each image, R was calculated for the original image as well as after randomization of pixels in one of the channels to quantify specificity of the co-localization.

#### Electrophysiology

Whole-cell voltage-clamp recording was performed on transfected cultured hippocampal neurons at DIV13-14 as described (Eckel et al., 2015, Smith et al., 2014, Davenport et al., 2017, Davenport et al., 2019). Neurons were held at −70 mV. Patch electrodes (4-5 MΩ) were filled with an internal solution containing (in mM): 120 CsCl, 5 QX314 Br, 8 NaCl, 0.2 MgCl_2_, 10 HEPES, 2 EGTA, 2 MgATP and 0.3 Na_3_GTP. The osmolarity and pH were adjusted to 300 mOsm/L and 7.2 respectively. The external artificial cerebro–spinal fluid (ACSF) solution consisted of the following (in mM): 125 NaCl, 25 NaHCO_3_, 2.5 KCl, 2 MgCl_2_, 1.25 NaH_2_PO_4_, 2 CaCl_2_, and 25 glucose (pH 7.4, 320 mOsm). This solution was supplemented with NBQX (20 μM), APV (50 μM) and TTX (1 μM) to isolate mIPSCs. All recordings were performed at room temperature (22-25°C). Series resistance (typically 10–20 mOhms) was monitored throughout the experiment. The access resistance, monitored throughout the experiments, was < 20 MΩ and results were discarded if it changed by more than 20%. Miniature events and their kinetics were analyzed using template-based event detection in Clampfit (version 10.5; Molecular Devices).

### Quantification and Statistical Analysis

For all quantified experiments the experimenters were blind to the condition of the sample analyzed, with the exception of mock transfected neurons (Figures 3 and S3). All experiments were performed at least 3 times from independent cell preparations and transfections, unless stated otherwise in the figure legend. Repeats for experiments are given in the figure legends as N-numbers and refer to number of cells of the respective conditions unless stated otherwise. Values are given as mean ± standard error of the mean (SEM). Error bars represent SEM. Statistical analysis was performed in GraphPad Prism (version 8; GraphPad Software, CA, USA) or Microsoft Excel. All data was tested for normal distribution with D’Agostino & Pearson test to determine the use of parametric (Student’s t test, one-way ANOVA) or non-parametric (Mann-Whitney, Kruskal-Wallis) tests. When p < 0.05, appropriate post hoc tests were carried out in analyses with multiple comparisons and are stated in the figure legends.

### Data and Code Availability

Datasets generated for this study are available from the corresponding author upon reasonable request. This study did not generate new code. A custom-made script using pre-existing tools in ImageJ to quantify antibody feeding experiments will be made available upon request.

## Supplemental Information

Document S1. Figures S1–S4

Document S2. Article plus Supplemental Information
