# Supplementary material for: SNX27-Mediated Recycling of Neuroligin-2 Regulates Inhibitory Signaling
Source: Cell Rep. 2019 Nov 26;29(9):2599–2607.e6. doi: 10.1016/j.celrep.2019.10.096 (PMC6899438; doi:10.1016/j.celrep.2019.10.096)
Supplement: Document S1. Figures S1–S4 [file mmc1.pdf]

Cell Reports, Volume *29*

Supplemental Information

SNX27-Mediated Recycling of Neuroligin-2 Regulates Inhibitory Signaling

Els F. Halff, Blanka R. Szulc, Flavie Lesept, and Josef T. Kittler

Figure S1

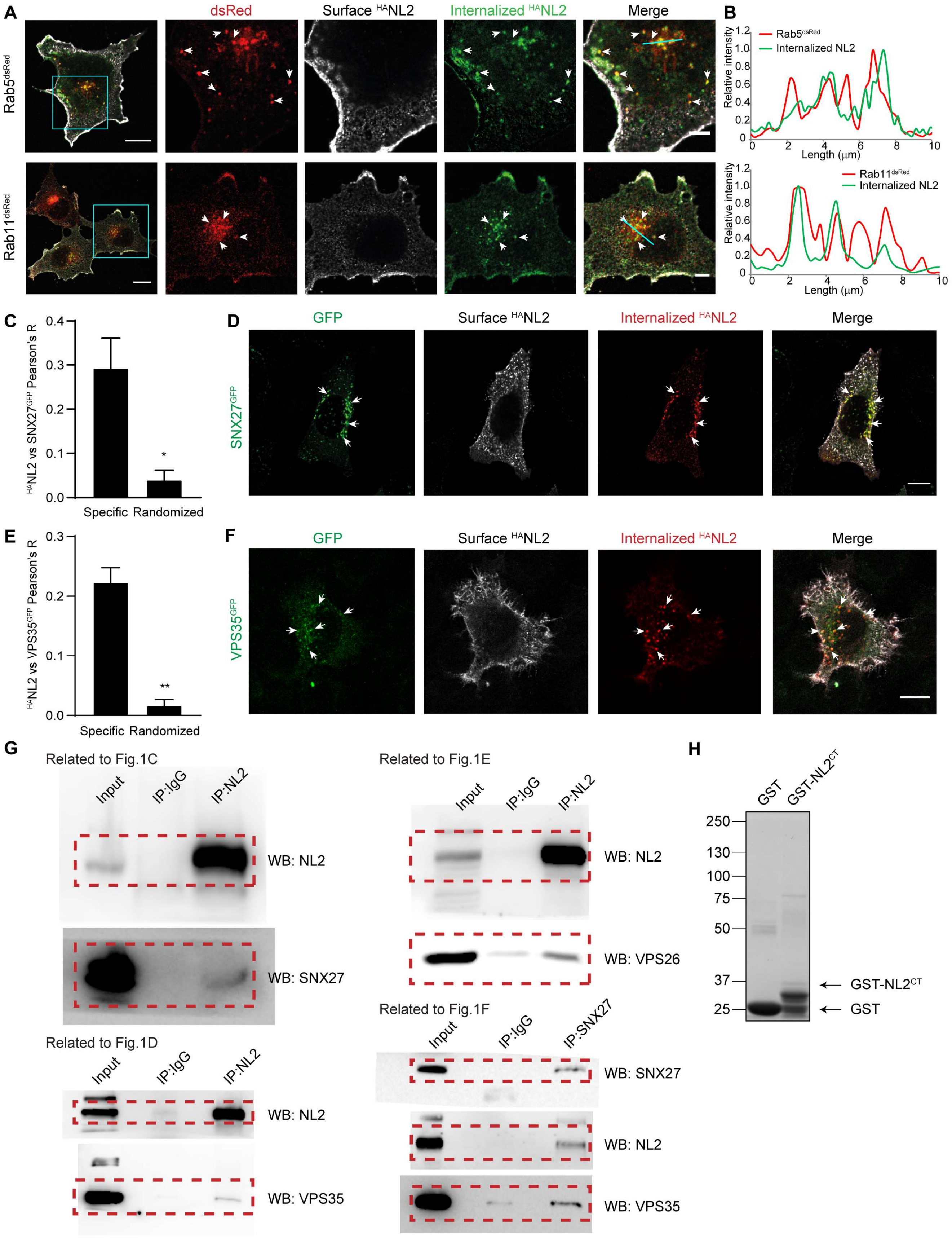

Figure S1 (related to Figure 1): Internalized NL2 co-localizes and interacts with endosomal markers and recycling complexes. A. Confocal images of antibody feeding in COS-7 cells showing co-localization of

internalized ^{HA}NL2 (green) with Rab5^{dsRed} (top) or Rab11^{dsRed} (bottom, red). Scale bars, 15 μm (whole cells) and 5 μm (zooms). Arrowheads show examples of co-localization. **B.** Example fluorescent intensity line scans through clusters (corresponding to the lines shown in the merged zoomed images in panel A), showing overlap between internalized ^{HA}NL2 and Rab5^{dsRed} or Rab11^{dsRed}. **C.** Pearson's coefficient of co-localization between ^{HA}NL2 and SNX27^{GFP} in neurons (related to Fig.1A; n=4 cells from 2 independent experiments). **D.** Confocal image of antibody feeding in HeLa cells showing co-localization of internalized ^{HA}NL2 (red) with SNX27^{GFP}. Arrowheads show examples of co-localization. Scale bar, 10 μm. **E.** Pearson's coefficient of co-localization between ^{HA}NL2 and VPS35^{GFP} in neurons (related to Fig.1B; n=3 cells from 1 experiment). **F.** Confocal image of antibody feeding in HeLa cells showing co-localization of internalized ^{HA}NL2 (red) with VPS35^{GFP}. Arrowheads show examples of co-localization. Scale bar, 10 μm. **G**. Full western blots related to the sections of western blots shown in Fig.1C-F. Red boxes indicate the part of the blot that was shown in Fig.1C-F. **H.** Coomassie gel of purified GST and GST-NL2^{CT}. Numbers on the left indicate molecular weight in kDa.

Values are mean $\pm$ SEM; *p<0.05, **p<0.01; paired two-tailed *t*-test.

Figure S2

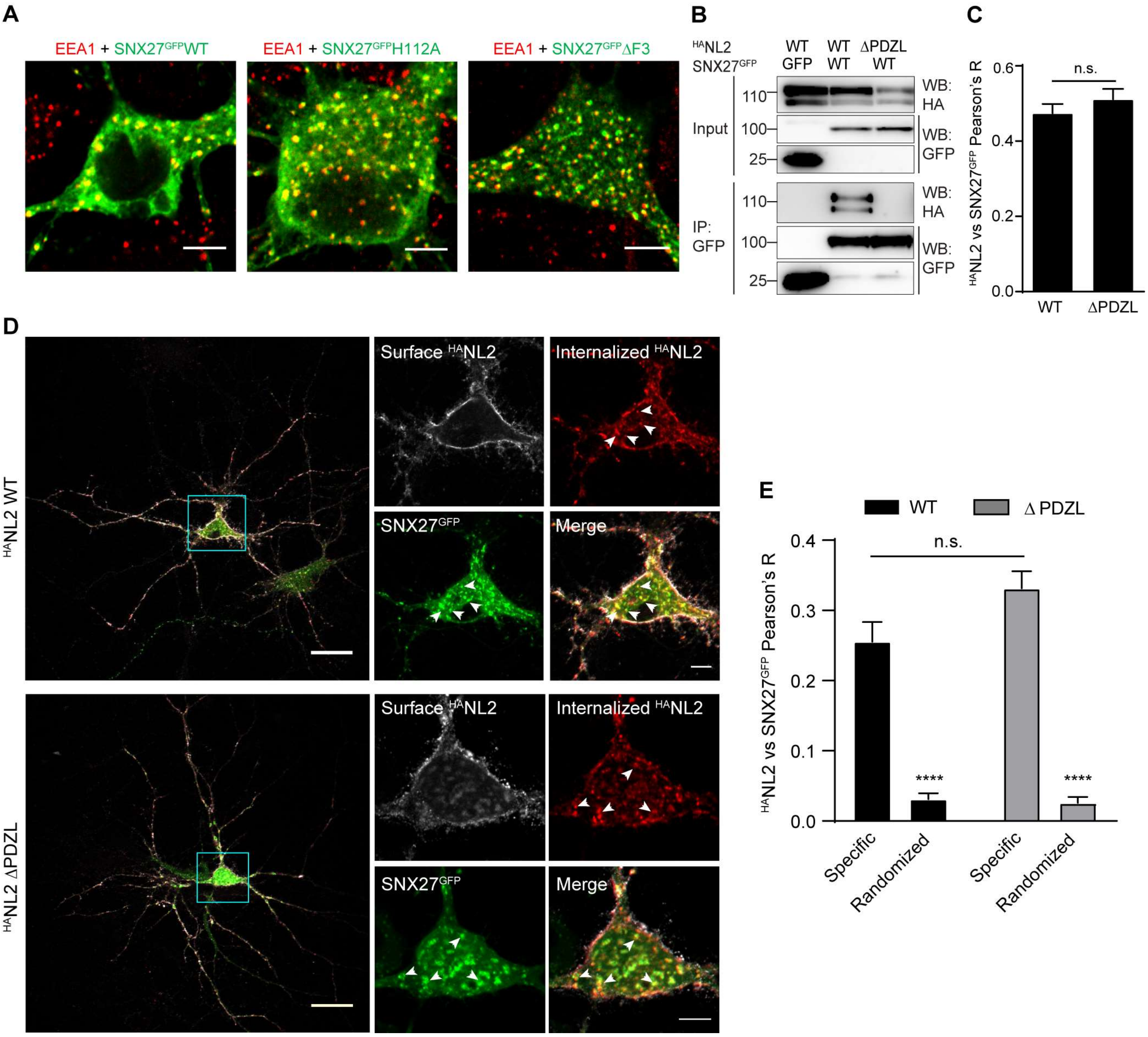

Figure S2 (related to Figure 2): Abolishing PDZ-ligand interaction between SNX27 and NL2 does not affect endosomal localization of SNX27 and NL2 mutants. A. Confocal images showing co-localization of WT and mutant SNX27^{GFP} with Early Endosome Antigen 1 (EEA1) in the soma of hippocampal neurons. Scale bar, 5 µm. **B.** Western blot showing co-immunoprecipitation from COS-7 cells co-expressing GFP-control with ^{HA}NL2WT (first lane) or SNX27^{GFP} with ^{HA}NL2 constructs (second and third lane). GFP and SNX27^{GFP} were pulled down using GFP-Trap beads (IP, immunoprecipitation). Numbers on the left indicate molecular weight in kDa. **C.** Pearson's coefficient of co-localization between SNX27^{GFP} and ^{HA}NL2WT or ^{HA}NL2ΔPDZL in HeLa cells (related to Fig. 2D; n=15 and 14 cells, respectively, from 2 independent experiments; unpaired two-tailed *t*-test). **D.** Confocal images of antibody feeding in hippocampal neurons showing co-localization of internalised ^{HA}NL2WT or ^{HA}NL2ΔPDZL (red) with SNX27^{GFP} (green) in the soma. Arrowheads show examples of co-localization. Scale bars, 25 µm (whole cells) and 5 µm (soma). **E.** Pearson's coefficient of co-localization between ^{HA}NL2WT or ^{HA}NL2ΔPDZL and SNX27^{GFP} in neurons (related to Fig.S2D; n=15 and 7 cells, respectively; two-way ANOVA with Bonferroni's correction).

Values are mean ± SEM; n.s. non-significant; ****p<0.0001.

Figure S3

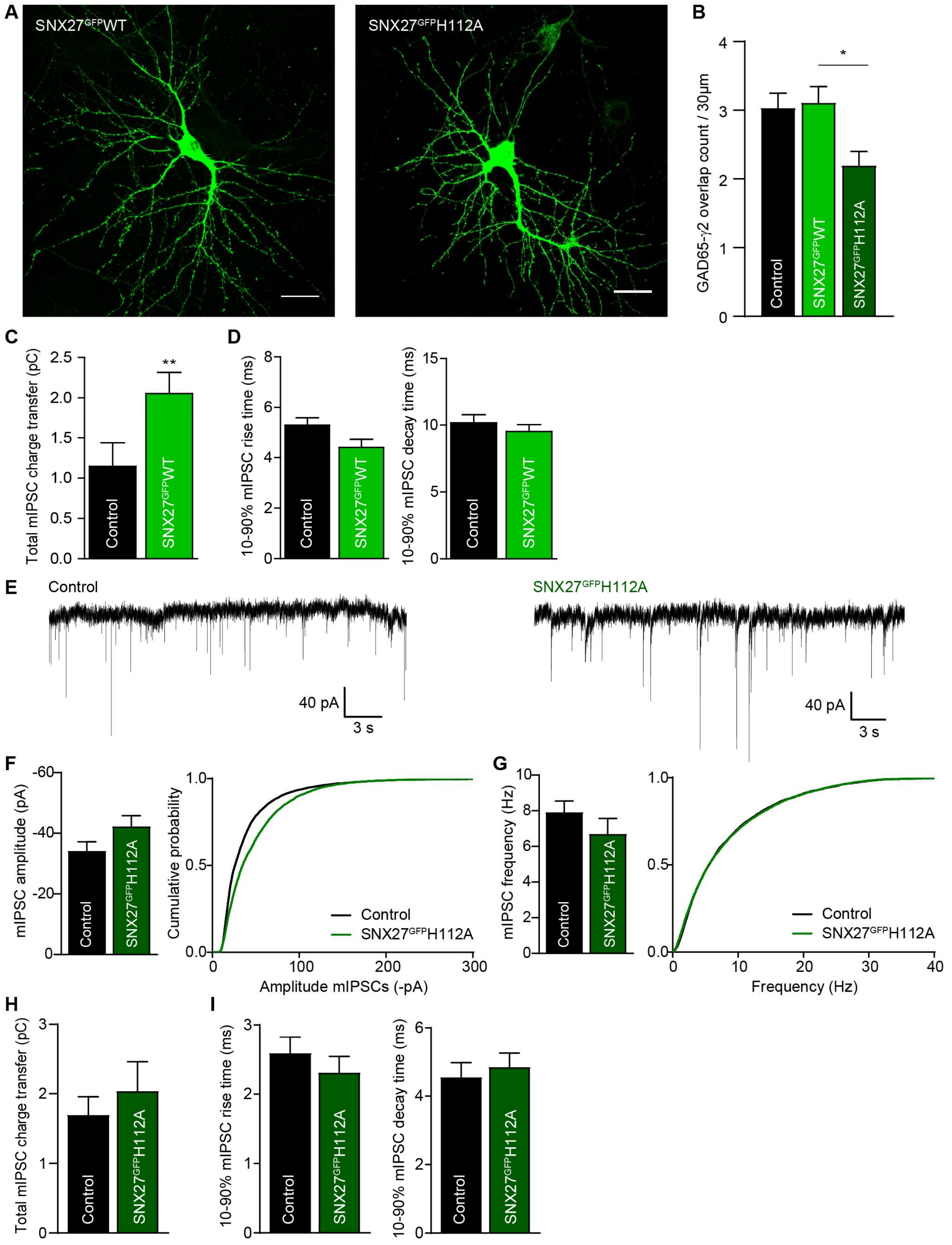

Figure S3 (related to Figure 3): Overexpression of SNX27^{GFP}WT but not SNX27^{GFP}H112A affects miniature IPSCs in hippocampal neurons. A. Confocal images of hippocampal neurons overexpressing SNX27^{GFP}WT (left) or SNX27^{GFP}H112A (right), showing its distribution throughout the neuron. Scale bar, 25 μm. **B.** Quantification of overlapping presynaptic GAD65 and postsynaptic γ2 clusters in hippocampal neurons overexpressing SNX27^{GFP}WT, SNX27^{GFP}H112A, or mock transfected (Related to Fig.3A-H; n=23,26,24 cells; Kruskal-Wallis test with Dunn's correction). **C.** Total charge transfer calculated for mIPSC of hippocampal

neurons that were mock transfected (Control), or overexpressing SNX27^{GFP}WT (WT) (related to Fig.3I-K; n=13 and 22 cells, respectively; Mann-Whitney test). **D.** Kinetics data related to whole-cell patch clamp recordings depicted in Fig. 3I-K. Rise time (Left) and decay time (Right) show no significant difference (n=13 and 22 cells, respectively; unpaired two-tailed *t*-tests). **E.** Representative traces of mIPSC patch-clamp recordings from hippocampal cultures, mock transfected (Control), or overexpressing SNX27^{GFP}H112A (H112A). **F-G.** Pooled data (left) and cumulative probability plot (right) of mIPSCs amplitude (**F**) and frequency (**G**) of hippocampal neurons that were mock transfected (Control), or overexpressing SNX27^{GFP}H112A (H112A) (n=11 cells each; unpaired two-tailed *t*-tests). **H.** Total charge transfer calculated for mIPSC of hippocampal neurons (related to panel E-G; unpaired one-tailed *t*-test). **I.** Kinetics data related to whole-cell patch clamp recordings depicted in panel E-G: rise time (Left; Mann-Whitney test) and decay time (Right; unpaired two-tailed *t*-test).

Values are mean $\pm$ SEM; *p<0.05, **p<0.01.

Figure S4

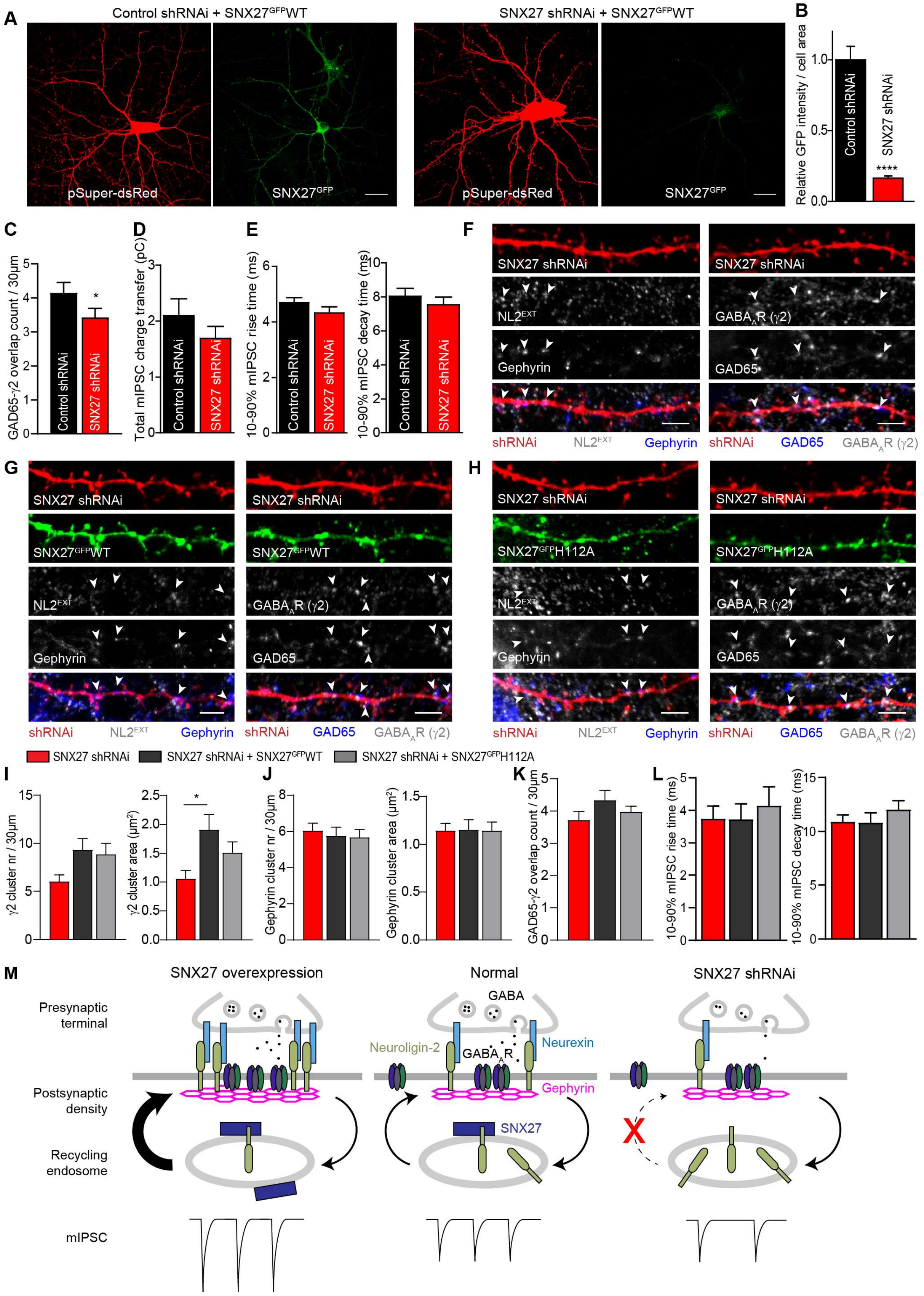
A
Control shRNAi + SNX27GFPWT
SNX27 shRNAi + SNX27GFPWT
pSuper-dsRed
SNX27GFP
B
Relative GFP intensity / cell area
Control shRNAi
SNX27 shRNAi
****
C
GAD65-γ2 overlap count / 30µm
D
Total mIPSC charge transfer (pC)
E
10-90% mIPSC rise time (ms)
10-90% mIPSC decay time (ms)
F
SNX27 shRNAi
NL2EXT
GABAAR (γ2)
Gephyrin
GAD65
shRNAi NL2EXT Gephyrin
shRNAi GAD65 GABAAR (γ2)
G
SNX27GFPWT
H
SNX27GFPH112A
SNX27 shRNAi + SNX27GFPWT
SNX27 shRNAi + SNX27GFPH112A
I
γ2 cluster nr / 30µm
γ2 cluster area (µm²)
J
Gephyrin cluster nr / 30µm
Gephyrin cluster area (µm²)
K
L
M
SNX27 overexpression
Normal
SNX27 shRNAi
Presynaptic terminal
Postsynaptic density
Recycling endosome
mIPSC
GABA
Neuroligin-2
Neurexin
GABAAR
Gephyrin
SNX27

Figure S4 (related to Figure 4): The effects of SNX27 knockdown can be rescued by SNX27^{GFP}WT but not SNX27^{GFP}H112A overexpression. A. Confocal images of hippocampal neurons transfected with control or SNX27-specific shRNAi (co-expressing dsRed), combined with murine SNX27^{GFP} (green) to assess the efficiency of knockdown. Scale bar, 25 µm. **B.** Quantification of relative SNX27^{GFP} intensity in hippocampal neurons, transfected as in panel A, shows a 84% decrease in GFP intensity with SNX27-specific shRNAi compared to control shRNAi (n=18 and 22 cells, respectively; unpaired two-tailed *t*-test). **C.** Quantification of overlapping presynaptic GAD65 and postsynaptic γ2 clusters in hippocampal neurons transfected with control or SNX27-specific shRNAi (related to Fig.4A-G; n=22 and 23 cells, respectively; unpaired one-tailed *t*-test). **D**. Total mIPSC charge transfer calculated for mIPSC of hippocampal neurons transfected with control or SNX27-specific shRNAi (related to Fig.4H-J; n=23 and 20 cells, respectively; unpaired two-tailed *t*-test). **E.** Kinetics data related to whole-cell patch clamp recordings depicted in Fig. 4H-J: rise time (left) and decay time (right) (n=23 and 20 cells, respectively; unpaired two-tailed *t*-tests). **F-H.** Confocal images of 30 µm dendritic sections of hippocampal neurons transfected with SNX27 shRNAi only (KD, **F**), or combined with RNAi-resistant SNX27^{GFP}WT (KD+WT, **G**) or SNX27^{GFP}H112A (KD+H112A, **H**). Neurons were stained for synaptic NL2 (NL2^{EXT}), GAD65, gephyrin, and the GABA$_A$R γ2 subunit. Arrowheads show synaptic clusters. Scale bar, 4 µm. **I-J.** Quantification of cluster number (left) and area (right) in hippocampal neurons transfected as in panel F-H. Staining is analysed for γ2 (**I**; n=23,22,24 cells; one-way ANOVA with Bonferroni's correction) and gephyrin (**J**; n=22,24,19 cells; cluster number, Kruskal-Wallis test; cluster area, one-way ANOVA). See Fig.4K-L for quantification of NL2^{EXT} and GAD65. **K.** Quantification of overlapping presynaptic GAD65 and postsynaptic γ2 clusters in hippocampal neurons transfected as in panel F-H (n=23,22,28 cells; one-way ANOVA). **L.** Kinetics of mIPSCs related to whole-cell patch clamp recordings depicted in Fig. 4M-P: rise time (left) and decay time (right) (n=16,15,13 cells; both one-way ANOVA). **M.** Schematic representation of the proposed molecular model, summarizing the data from Fig.3 and Fig.4, and explaining how SNX27-mediated recycling of NL2 modulates inhibitory signaling. Increased SNX27 activity and NL2 recycling enhances stabilization of the postsynaptic scaffold via recruitment of gephyrin and GABA$_A$Rs, resulting in increased mIPSC amplitude. SNX27 knockdown and impaired NL2 recycling destabilizes the presynaptic terminal, leading to reduced mIPSC frequency.

Values are mean ± SEM; *=p<0.05, **=p<0.01, ***=p<0.001, ****=p<0.0001.
